# Clinical Therapy in Dementia and Related Diseases

**DOI:** 10.3390/jcm15124526

**Published:** 2026-06-11

**Authors:** Takao Yamasaki

**Affiliations:** 1 Department of Neurology, Minkodo Minohara Hospital, Fukuoka 811-2402, Japan; yamasaki@kyudai.jp; Tel.: +81-92-947-0040; 2 Department of Research and Development, Kumagai Institute of Health Policy, Fukuoka 816-0812, Japan

## 1. Introduction

The global rise in dementia prevalence continues to impose profound clinical, societal, and economic challenges [1]. Despite substantial progress in recent years, key limitations remain, including the modest and stage-dependent efficacy of current disease-modifying therapies targeting amyloid pathology [2], the complexity of early diagnosis, and the persistent gap between mechanistic insights and clinical application.

Dementia is increasingly recognized as a condition that cannot be adequately understood through isolated, single-domain perspectives, but rather as one emerging from the interaction of multiple biological and systemic processes. As such, there is a growing need for integrative frameworks that capture these interdependencies and enable their translation into meaningful therapeutic strategies.

This Special Issue, “Clinical Therapy in Dementia and Related Diseases”, was conceived in response to this need. The contributions collectively reflect a shift from reductionist approaches toward a systems-level understanding of dementia, in which diverse domains are treated as interconnected components of a continuous translational spectrum.

Specifically, the six contributions illustrate how advances can be aligned across multiple levels, linking molecular mechanisms, brain network function, real-world behavior, technological innovation, and system-level care. Importantly, these domains are not sequential but mutually interactive: molecular dysfunction shapes network dynamics; network disruption manifests as functional impairment; and clinical needs drive technological and system-level innovation. Conversely, interventions at the level of care delivery and real-world environments may influence disease trajectories.

By organizing these perspectives within a unified conceptual framework, this Special Issue aims to bridge the gap between mechanistic discovery and clinical implementation, providing a foundation for integrated, multi-level strategies to improve outcomes in dementia care.

## 2. Molecular Mechanisms and Therapeutic Targets

At the foundation of this framework lies molecular pathophysiology and its translation into therapeutic innovation. Takahashi and Muguruma (Contribution 1) present an integrative view of Alzheimer’s disease that shifts the focus beyond traditional plaque-centric models toward intracellular amyloid-β toxicity and endo-lysosomal dysfunction as central organizing mechanisms.

Rather than treating amyloid-β, tau, and neuroinflammation as independent pathological features, their synthesis highlights the endo-lysosomal system as a critical hub linking protein aggregation, lipid metabolism, and cellular stress responses. Within this framework, Alzheimer’s disease emerges as a disorder of disrupted intracellular homeostasis, in which tightly coupled feedback loops amplify neurodegenerative processes across molecular pathways.

This perspective is particularly relevant in the context of current disease-modifying therapies targeting amyloid-β, whose modest and stage-dependent efficacy underscores the limitations of single-target approaches [3]. By reframing Alzheimer’s disease as a network of interacting molecular systems, this work provides a conceptual basis for multi-target and combination strategies that extend beyond amyloid reduction alone.

Ultimately, this contribution illustrates a broader shift in the field—from targeting isolated pathological hallmarks to modulating interconnected biological systems—as a prerequisite for achieving more effective and durable therapeutic outcomes.

## 3. Network-Level Brain Dynamics as a Translational Bridge

Building upon the molecular perspective, Chuda et al. (Contribution 2) extend the discussion to the level of large-scale brain network dynamics. Their longitudinal findings demonstrate that functional reorganization within key intrinsic networks—including the default mode network and salience-related regions—can occur rapidly following clinical intervention, even when the intervention itself is not primarily neurological.

These observations highlight a critical insight: network-level dysfunction in conditions such as chronic pain and dementia may not represent fixed deficits, but rather dynamic and potentially reversible states. Disruptions in large-scale network integration, particularly involving interactions between internally oriented and salience-processing systems, are increasingly recognized as a shared feature across diverse brain disorders [4].

Importantly, the study provides a conceptual framework for understanding how peripheral, cognitive, and affective processes converge at the level of brain networks. The dissociation between sensory and cognitive–emotional components further suggests that network dynamics may be modulated through multiple pathways, not limited to primary disease mechanisms.

Taken together, this work positions large-scale brain networks as a key translational bridge linking biological processes to clinical outcomes, and underscores their potential as targets for interventions aimed at modifying disease-relevant functional states.

## 4. Functional Capacity and Real-World Behavior

At the level of functional capacity and real-world behavior, Tanaka et al. (Contribution 3) provide important insights into the mechanisms underlying everyday performance in older adults. Their work highlights a load-dependent breakdown of compensatory processes: while performance may be preserved under low-to-moderate demands, it becomes unstable as cognitive load increases.

This finding offers a compelling explanation for the discrepancy often observed between preserved abilities in structured settings and failures in complex real-world environments [5]. Daily activities such as driving require rapid adaptation to unpredictable and high-demand situations, where latent vulnerabilities may emerge despite apparently intact baseline performance.

Importantly, this perspective shifts the focus from static assessments of ability to condition-dependent evaluation. Functional capacity is not fixed, but varies according to task demands and environmental complexity. As such, conventional assessments may underestimate risk if they fail to capture the conditions under which compensatory mechanisms break down.

Taken together, this work underscores the need for ecologically valid and load-sensitive frameworks for evaluating real-world function, particularly in aging populations and early stages of cognitive decline.

## 5. Digital Innovation and Non-Pharmacological Interventions

The integration of digital technologies into dementia care represents a promising avenue for non-pharmacological intervention. Eckert et al. (Contribution 4) demonstrate the feasibility and acceptability of immersive virtual reality in individuals with dementia, highlighting a shift from task-based training toward exploratory and experience-driven environments.

This approach is notable in that it engages multiple domains simultaneously, including cognition, motor function, and affective processing. By emphasizing open-ended interaction rather than performance optimization, immersive environments may better reflect the complexity of real-world behavior and provide more ecologically valid forms of stimulation [6].

Importantly, these findings suggest that digital interventions can move beyond compensatory support toward the modulation of underlying functional systems. However, their clinical impact remains to be established, and further studies are required to determine their efficacy, durability, and scalability across diverse care settings.

From a broader perspective, this work illustrates how technological innovation can serve as a bridge between experimental neuroscience and real-world care. When integrated into clinical and community frameworks, such approaches have the potential to expand the scope of personalized, accessible, and engaging interventions in dementia care.

## 6. Personalized Medicine and Predictive Analytics

Advancing from intervention toward individualized optimization, Nakahara et al. (Contribution 5) highlight the potential of predictive analytics in guiding treatment strategies. By applying machine learning to integrate pharmacological and clinical variables, their work addresses a central challenge in neuropsychiatric care: the substantial variability in treatment response across individuals.

Although focused on treatment-resistant schizophrenia, this framework has clear relevance for dementia and related disorders, where heterogeneity in disease progression and therapeutic response remains a major barrier to effective care. The ability to anticipate treatment outcomes and tailor interventions accordingly represents a critical step toward precision medicine in complex brain disorders [7].

Importantly, this approach shifts clinical decision-making from reactive adjustment to proactive prediction. Rather than relying on trial-and-error strategies, predictive models offer the potential to align therapeutic choices with individual profiles from the outset.

Within the broader context of this Special Issue, such analytical frameworks provide a key link between biological complexity and clinical application, supporting the development of more targeted, efficient, and personalized treatment strategies.

## 7. Integrated Care Systems and Clinical Implementation

Finally, Yamasaki (Contribution 6) addresses the system-level dimension of dementia care by proposing an integrated model that aligns clinical practice across settings. By bridging outpatient and inpatient care and facilitating interdisciplinary collaboration, this framework emphasizes continuity, coordination, and person-centered care as essential components of effective dementia management.

Importantly, this contribution highlights that advances in diagnostics and therapeutics cannot achieve meaningful impact in isolation. Even the most sophisticated interventions depend on healthcare systems capable of delivering them consistently across diverse and often fragmented care environments [1]. In this context, general medicine physicians are positioned as key integrators, linking medical treatment with functional, social, and environmental support throughout the disease trajectory.

This perspective reframes implementation not as a logistical challenge, but as a core determinant of clinical effectiveness. The success of emerging therapies will depend not only on biological efficacy, but also on whether care systems can accommodate complexity, multimorbidity, and long-term needs.

Taken together, this work underscores a central message of this Special Issue: innovation in dementia care must extend beyond discovery to include the design of systems capable of translating advances into real-world outcomes.

## 8. Toward an Integrated Framework

Building on the multi-level perspectives presented above, this Special Issue collectively supports a reconceptualization of dementia as a multi-level, dynamically interacting system. Dementia is not adequately explained as a linear cascade of pathological events, but as an emergent condition arising from continuous interactions across molecular, network, behavioral, and healthcare domains.

Within this framework, processes at different levels are tightly coupled. Molecular and cellular disturbances shape large-scale brain network organization; network-level alterations manifest as impairments in real-world function; and these clinical realities drive the development of technological innovations and care systems. Conversely, interventions at the level of behavior, environment, and care delivery may influence underlying biological processes.

This perspective shifts the focus from isolated targets to system-level modulation. Therapeutic strategies—ranging from molecular interventions to digital technologies and integrated care models—should therefore be understood as complementary components within a unified therapeutic architecture.

The central contribution of this Special Issue lies in demonstrating the necessity of aligning these domains into a coherent and actionable framework for both research and clinical practice.

## 9. Remaining Challenges

Despite this conceptual advance, several challenges remain for translating this framework into clinical impact.

First, the integration of findings across biological and functional scales remains limited. Mechanistic links between molecular pathology, network dysfunction, and real-world outcomes are not yet fully established [8], requiring longitudinal and multimodal approaches.

Second, the translational validity of emerging biomarkers and therapeutic targets remains uncertain in heterogeneous real-world populations, where comorbidity and environmental factors introduce additional complexity.

Third, while digital and non-pharmacological interventions are promising, robust evidence regarding their efficacy, durability, and scalability is still needed.

Finally, the implementation of integrated care systems presents practical challenges. Conceptual models must be supported by empirical validation, workforce development, and alignment with healthcare structures to ensure sustainability.

Addressing these challenges will require coordinated efforts that extend beyond individual domains toward truly integrative strategies.

## 10. Future Directions and Conclusions

Looking ahead, progress in dementia care will depend on integrative and translational approaches that align advances across biological, functional, technological, and system-level domains.

This includes integrating multimodal biomarkers, developing ecologically valid assessment frameworks, designing scalable digital interventions, and strengthening coordinated models of care that ensure continuity across settings. Importantly, progress will depend not only on advances within each domain, but on their effective integration into actionable strategies.

In this context, combination approaches that link molecular targeting, network modulation, functional support, and optimized care delivery [1] may offer the greatest potential for meaningful clinical impact.

In conclusion, this Special Issue reflects a shift from reductionist perspectives toward a systems-level understanding of dementia. By connecting advances across multiple domains, it provides a foundation for integrated strategies capable of addressing the complexity of the disease.

As Guest Editor, I hope this work stimulates further interdisciplinary collaboration and contributes to the development of more effective and scalable approaches to dementia care.
